# Susceptibility of Opportunistic *Burkholderia glumae* to Copper Surfaces Following Wet or Dry Surface Contact

**DOI:** 10.3390/molecules19079975

**Published:** 2014-07-09

**Authors:** Zhouqi Cui, Muhammad Ibrahim, Chunlan Yang, Yuan Fang, Hussain Annam, Bin Li, Yanli Wang, Guan-Lin Xie, Guochang Sun

**Affiliations:** 1 State Key Laboratory of Rice Biology, Institute of Biotechnology, Zhejiang University, Hangzhou 310058, China; E-Mails: twelue@163.com (Z.C.); clyang0202@163.com (C.Y.); glxie@zju.edu.cn (G.-L.X.); 2 Department of Biosciences, COMSATS Institute of Information Technology, Sahiwal 57000, Pakistan; E-Mails: ibrahim@ciitsahiwal.edu.pk (M.I.); anam.hussain63@yahoo.com (H.A.); 3 College of Chemistry and Life Sciences, Zhejiang Normal University, Jinhua 321004, China; E-Mail: fy0579@zjnu.cn; 4 Zhejiang Academy of Agricultural Sciences, Hangzhou 310021, China; E-Mail: ylwang88@aliyun.com

**Keywords:** *B. glumae*, copper, antibacterial activity, membrane damage

## Abstract

*Burkholderia* *glumae* has been proposed to have a potential risk to vulnerable communities. In this work, we investigated the antibacterial activity and mechanism of copper surfaces against multi-drug resistant *B. glumae* from both patients and rice plants. The susceptibility of *B. glumae* to copper surfaces was noted by a significant decline in viable bacterial counts, relative to the slight reduction of stainless steel and polyvinylchloride, both of which were used as control surfaces. The mode of action of bacterial killing was determined by examing the mutagenicity, DNA damage, copper ions accumulation, and membrane damage in bacterial cells. The results indicated that the cells exposed to copper surfaces did not cause severe DNA lesions or increase the mutation frequencies, but resulted in a loss of cell membrane integrity within minutes. Furthermore, bacterial cells exposed to copper surfaces accumulated significantly higher amounts of copper compared to control surfaces. Overall, this study showed that metallic copper had strong antibacterial effect against *B. glumae* by causing DNA and membrane damage, cellular accumulation of copper, and cell death following DNA degradation*,* which could be utilized to reduce the risk of bacterial contamination and infection.

## 1. Introduction

*Burkholderia glumae* has been reported to be able to colonize several environmental niches and to cause chronic infections in humans [1,2,3,4,5,6,7,8]. Furthermore, it is also very difficult to differentiate the human strains from the environmental strains, although only several cases of human infection were identified. This indicated that these strains of different origin might be sharing a similar pattern of virulence. Currently, special attention has been paid to the possible role of environmental strains in human infections [9]. Indeed, the ubiquitous occurrence of street foods in most Asian countries poses a high risk of contamination with opportunistic *B. glumae*, resulting in food borne epidemics [10], while door knobs and other tactile surfaces in hospitals have been considered to be notorious primary sources of nosocomial infection due to contamination by opportunistic pathogens that can cause fatalities in intensive care units or nurseries [11]. In addition, these bacterial strains often exhibited multi-drug resistance, making clinically relevant antimicrobials ineffective [12]. Therefore, it is necessary to find an alternate method to inhibit the spread of these multi-drug resistant bacteria.

Copper is an indispensable element of aerobic metabolism that works as a cofactor, however, in low amounts and in excess, it can have deleterious effects [13]. The ability of copper to alternate between cuprous Cu (I) and cupric (II) ions, and its oxidation states have been considered to be the basis of copper toxicity [13]. The use of copper by human civilizations goes back to the 6th and 5th millennia [14], however, it was not until 2008 that its efficiency to inactivate microbes upon contact was identified [15,16,17,18]. After rigorous testing, copper alloys have been registered by the US Environmental Protection Agency as an antimicrobial agent.

The purpose of this study was to determine the antibacterial effect and mechanism of copper surface against opportunistic *B. glumae* based on viable bacterial counts, and the assays of mutagenicity, DNA damage, copper ions accumulation, and membrane damage in bacterial cells.

## 2. Results and Discussion

### 2.1. Susceptibility of B. glumae to Copper Surfaces

**Figure 1:**
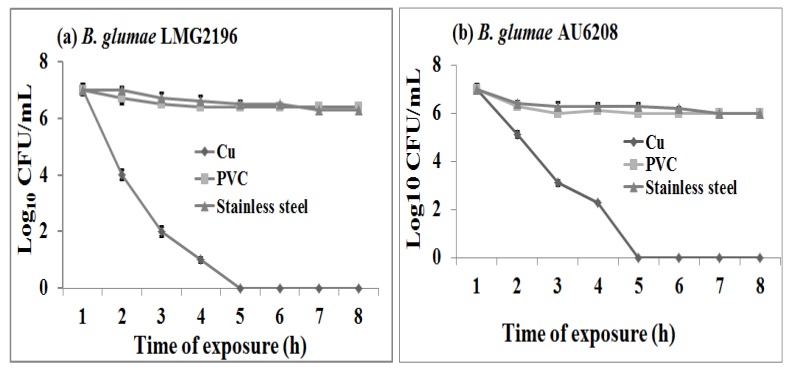
Survival of *Burkholderia glumae* strains (**a**) LMG2196 and (**b**) AU6208 on copper, PVC and steel surfaces, respectively, within 8 h of contact time. The initial concentration of bacterial suspensions was 5 × 10^7^ CFU/mL. The plots showed a significant difference (*p <* 0.05) in the logarithms of the surviving bacterial counts (CFU/mL) between copper and control surfaces regardless of the bacterial strains.

The viability assays were performed to examine the antibacterial activity of copper contact killingagainst *B. glumae*. The results in this study indicated that the polyvinylchloride (PVC) or stainless steel surfaces had no, or very slight, antibacterial activity at room temperature against both strains of *B. glumae*. On these materials, the reduction of viable bacterial counts varied from 1.2 to 1.5 log_10_ CFU/mL within 8 h of contact time compared to the initial bacterial concentration of approximately 5 × 10^7^ CFU/mL (Figure 1). However, compared to the PVC or stainless steel surfaces, the metallic copper surfaces showed strong antibacterial activity against *B. glumae* at room temperature regardless of the bacterial strains and the contact time (*p* *<* *0.05*). To the best of our knowledge, this is the first report about the antibacterial activities of copper surfaces against the multi-drug resistant * B. glumae* strains.

Result from this study indicated that the reduction in viable bacterial counts of *B. glumae* increased with the increase of contact time regardless of the bacterial strains. Indeed, after exposure of bacterial cells to copper surfaces at room temperature, there was a 0.7, 3.5, 5.5, and 6.6 log_10_ CFU/mL reduction in bacterial numbers of strain AU6208, while a 0.7, 3.4, 5.5, and 6.5 log_10_ CFU/mL reduction in bacterial numbers of strain LMG2196 after 1 h, 2 h, 3 h, and 4 h of contact time compared to the initial value, while no viable bacterial cells of both strains were found beyond 5 h of contact time between copper coupons and *B. glumae* (Figure 1). Interestingly, many copper tolerance genes such as *copA*, *copB*, *copC*, *copD*, *copM*, *cueR*, and *cueO* have been reported in some gram-positive and gram-negative bacteria. However, only *copC* gene was found in *B. glumae* AU6208 and LMG2196, based on BLAST analysis of their genomic sequences.

The result showed that the reaction of rice strain LMG2196 to PVC, stainless steel and metallic copper surfaces was similar to human strain AU6208, revealing that both strains might be sharing a similar response pattern to metals although it is still unclear about the role of rice strain in human infection. However, some of bacterial infections occurring in patients are likely through environmental exposure and food chain. Furthermore, the surveillance of foodborne disease outbreaks suggests that the infections are mostly due to the following significant contributing factors such as food from unsafe sources, inadequate cooking, improper holding temperatures, contaminated equipment, and poor personal hygiene. However, results in this study revealed the antibacterial effect of metallic copper surfaces against both human and rice strains, which directly and indirectly reduced the infection risk of *B. glumae* to humans.

Our study revealed that the exposure time significantly affected the antibacterial activity of metallic copper surfaces against *B. glumae* strains LMG2196 and AU6208, which was consistent with the result of some other bacterial species [15,19]. Indeed, the inhibitory effect of copper surfaces against *Salmonella enterica* and *Campylobacter jejuni* has been reported to be dependent on the exposure time of bacterial suspensions, while during the exposure of bacteria to copper sheets, the bacterial suspensions acquired a pale blue color that was indicative of the release of Cu^++^ ions and this color became more intense over time [15]. Therefore, it could be suggested that the exposure time may be also one of the vital factors contributing to bacterial killing of copper surfaces against *B. glumae*.

### 2.2. Acute Toxicity of Copper to B. glumae

**Figure 2:**
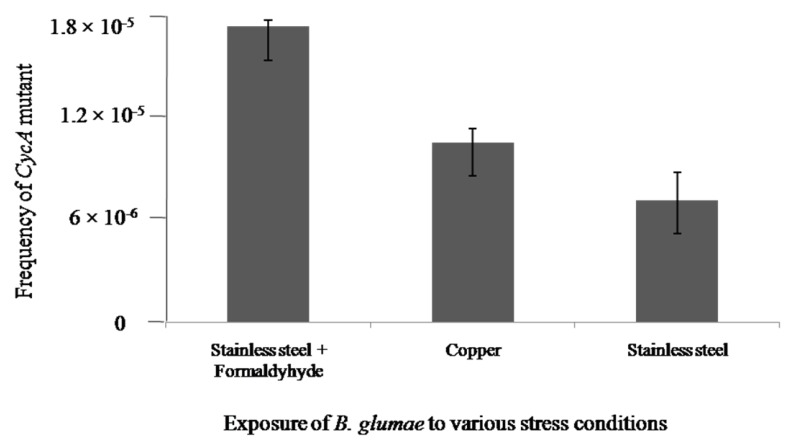
The frequencies of *cycA* mutants in *B. glumae* strains exposed to metallic copper, stainless steel, or stainless steel in the presence of formaldehyde, respectively.

Mutagenesis assay was conducted in this study to examine the acute toxicity of copper to *B. glumae*. Figure 2 presents the frequencies of mutation when bacterial cells at the concentration of 5 × 10^7^ CFU/mL were exposed to stainless steel, dry copper surfaces, or stainless steel in the presence of formaldehyde, respectively. In general, the D-Cycloserine based mutagenicity assay indicated that the exposure of dry copper surfaces did not significantly increase the mutation frequency in both strains of *B. glumae* compared to stainless steel. However, there was a significant increase in the mutation frequency when bacterial cells were exposed to stainless steel in the presence of formaldehyde, which is a known mutagen. This result of mutagenicity assays indicated that dry copper surfaces had weak genotoxic effect on *B. glumae*.

### 2.3. DNA Damage

**Figure 3:**
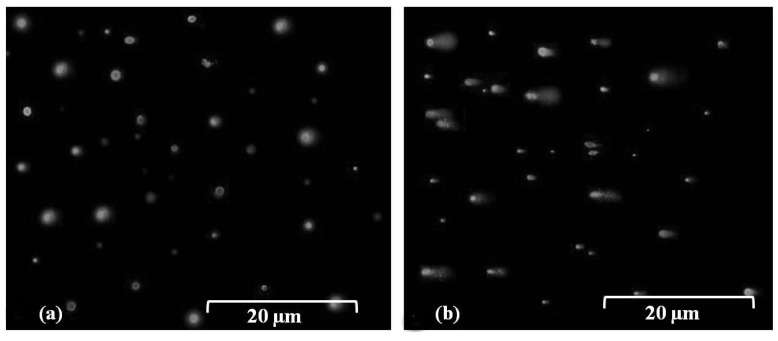
SGCE assay for the DNA damage of bacterial cells after 30 s exposure of *B. glumae* strains to (**a**) PVC control surfaces. (**b**) Dry metallic copper surfaces. Bar = 20 μm.

Comet assay or single cell gel electrophoresis (SCGE) were carried out in this study to examine DNA damage in bacterial cells. Results indicated that no tail or DNA fragmentation was observed when the cells of *B. glumae* were exposed for 30 s to stainless steel and PVC surfaces (Figure 3a). However, the comet tail was visualized after exposure of *B. glumae* cells to dry metallic copper surfaces for 30 s based on gel electrophoresis of SGCE assay, showing DNA damage in bacterial cells (Figure 3b). Furthermore, these results indicated that DNA was lethally damaged after 60 s of exposure to dry metallic copper surfaces, which is the time to kill bacteria or to induce acute toxicity, while no lethal effects occurred at 30 s of exposure (data not shown). Results of our comet assay indicated that exposure of *B. glumae* to dry copper surfaces, for short or extended periods of time, caused DNA damage that likely precedes cell death. These findings are consistent with the result of Espirito-Santo *et al.* [13], who demonstrated that exposure of *Escherichia coli* to dry metallic copper surfaces resulted in toxicity due to DNA damage following prolonged exposure.

### 2.4. Accumulation of Copper Ions in Cells

**Figure 4:**
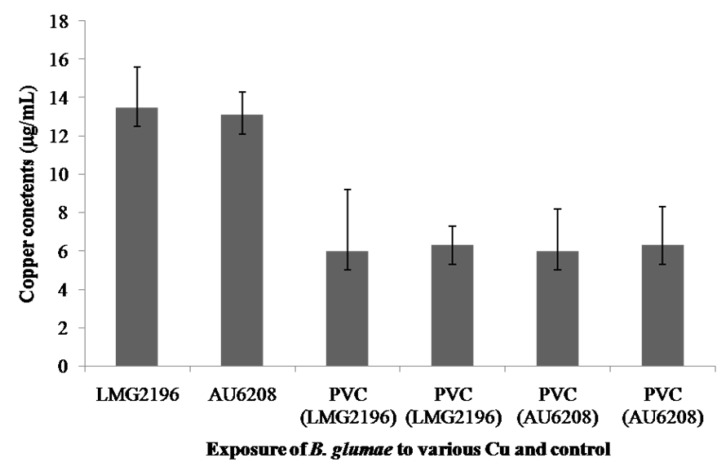
The accumulation of copper in *B. glumae* cells. The copper contentswere measured after 3 h of exposure to metallic copper surfaces, PVC, and stainless steel, respectively.

The influx of copper ions into bacterial cells was assayed in this study to examine the accumulation of copper ions in *B. glumae*. Indeed, the copper contents of *B. glumae* LMG2196 and *B. glumae* AU6208 were 13.5 µg/mL and 13.1 µg/mL, respectively, when both *B. glumae* strains were exposed to metallic copper surfaces for 3 h, while the average copper contents of *B. glumae* LMG2196 and *B. glumae* AU6208 were 6.0 µg/mL and 6.3 µg/mL, respectively, when both strains were exposed to PVC and stainless steel for 3 h, respectively (Figure 4).

In general, results from this study indicated that copper accumulation in *B. glumae* cells exposed to metallic copper surfaces was significantly (*p* *<* 0.05) increased compared to that exposed to PVC and stainless steel based on the assay of ICP-MS (Figure 4). The result indicated that exposure of *B. glumae* strains LMG2196 and AU6208 to metallic copper surfaces caused an accumulation of copper in bacterial cells compared to that of PVC and stainless steel, which was consistent with the antibacterial effect of metallic copper surfaces against the *B. glumae* strains. Furthermore, Espirito-Santo *et al.* (2011) have revealed that copper accumulation in bacterial cells is the key factor resulting in copper toxicity and subsequent cell death in *E. coli* [13]. Therefore, it could be suggested that the contact killing of *B. glumae* cells was, at least partially, due to the accumulation of copper, while free copper ions are likely to cause a selective change in bacterial membrane, which is the direct target of copper exposure [13,20,21]. Interestingly, the data obtained in this study from ICP-MS demonstrated the correlation between the increased concentration of copper in bacterial cells, as shown in Figure 4, and bacterial killing by metallic copper surfaces, as shown in Figure 1.

### 2.5. Damage in Cell Membranes

**Figure 5:**
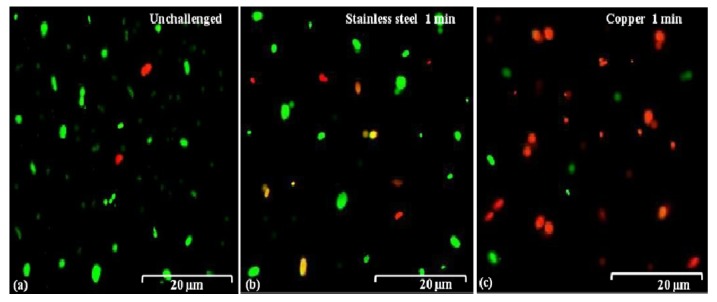
Membrane damage of *B. glumae* cells exposed to copper surfaces for 60 s. Staining were carried out using live/dead *Bac*Light bacterial viability kit (Invitrogen), and visualized by fluorescence microscopy. Fluoresce green is representative of live bacteria with intact membranes, while fluoresce red is representative of the damaged membranes.

The action mode of bacterial killing was further determined by examing the membrane damage of *B. glumae* cells exposed to copper surfaces. In this study, bacterial cells were removed from the coupon surface with PBS buffer after exposure to dry copper coupons for both 30 and 60 s, and live/dead staining based on SYTO 9 and propidium iodide, and then immediately subjected to fluorescence microscopy. The SYTO 9, when used alone, labels both live and dead bacterial DNA, while propidium iodide labels only bacteria with damaged membranes, leading to the reduction of SYTO 9 fluorescence in the presence of both dyes. As a result, live bacteria with intact membranes produce fluoresce green and bacteria with damaged membranes produce fluoresce red. In general, this result indicated that there was no significant difference in membrane damage between both strains, when bacterial cells were exposed to metallic copper, stainless steel, and PVC. Indeed, cells of both strains, exposed to stainless steel or PVC, remained largely green and, thus, undamaged, which was assessed by use of a dye, propidium iodide that enters cells and stains cellular DNA only if the cellular membranes are damaged (Figure 5a,b). However, most cells of both strains turned red when bacteria were exposed to copper surfaces for longer time periods, indicating a damage of membrane structure (Figure 5c). Therefore, it seems that membrane damage of *B. glumae* cells contributes to the action mode of metallic copper.

In agreement with the result of this study, previous studies have revealed that bacterial killing by copper is preceded by successive membrane damage, copper influx into the cells, oxidative damage, and DNA damage [13]. Furthermore, some studies have demonstrated the antimicrobial effect of copper on a range of disease-causing organisms, as reviewed by Grass *et al.* [19]. However, to the best of our knowledge, no information is available concerning the antibacterial activity of copper against the clinical or environmental isolates of *B. glumae* and the role of cellular membrane in susceptibility of *B. glumae* strains to copper. Overall, this study could provide important information about the applications of copper against this important opportunistic pathogen in hospitalized and immunocom promised patients, as well as in food processing industries.

## 3. Experimental

### 3.1. Bacterial Strains and Culture Conditions

*B. glumae* strain LMG2196, reported as a rice panicle pathogen, was acquired from Prof. Swing, BCCM/LMG Bacteria Collection at the University of Ghent, Belgium. Furthermore, Prof. LiPuma from the USA provided us the *B. glumae* strain AU6208, which has been reported to cause chronic granulomatous disease (CGD) [9]. Both strains of *B. glumae* were stored in 20%–30% glycerol (Shanglin Industries, Hangzhou, China) at −80 °C and were cultured for 24–48 h in Brain Heart Infusion (BHI) agar at 37 °C. Single colonies were inoculated in 20 mL BHI broth and incubated at 37 °C overnight; on the following day, cultures were diluted with 10 mL fresh BHI in 50 mL flasks to an approximate optical density (OD) at 600 nm of 2.0, which was determined using a spectrophotometer (Perkin Elmer Lambda35 UV/VIS).

### 3.2. Metallic Copper Surfaces

Metallic copper coupons used in this study were purchased from Teachn Industrial Technology Development Co., Limited, Hunan, China. They were cut to the size of 1.5 cm × 1.5 cm × 0.5 mm. Stainless steel sheets (0.5 mm) and polyvinylchloride (PVC), obtained locally, were used as controls.

### 3.3. Viability Assays

The antibacterial activity of copper contact killing against *B. glumae* was evaluated using suspensions of strains LMG2196 and AU6208. The viability assays were performed, as described by Mehtar *et al.* [22], with minor amendment. Bacterial culture was serially diluted in phosphate buffer saline (PBS), and 20 μL of bacterial suspensions (5 × 10^7 ^CFU/mL) were overlaid on sterile copper surfaces, PVC or stainless steel surfaces, respectively, and then were stored at room temperature as described by Ibrahim *et al.* [23]. The viable bacterial counts were measured at 1, 2, 3, 4, 5, 6, 7, and 8 h to determine the effect of various time intervals on the antibacterial activity of copper surfaces against both strains of *B. glumae*. Copper, stainless steel, and PVC coupons were kept in Petri dishes that were maintained in a closed plastic box. Whatman filter paper was used inside the walls of the box and was kept wet with sterile distilled water for different times until completion of the experiment. After incubations of different times, coupons were placed in 10 mL of PBS with 2 mm glass beads (PBSG), centrifuged for 30 s at 300 *×g*, and then serially diluted to measure the bacterial count. The assays were performed in duplicate with three replications.

### 3.4. Mutagenicity Assay

Mutagenesis assay was conducted as described by Tian *et al.* [24]. Briefly, bacterial cells were applied for 60 s (an exposure period shorter than that required for massive onset of lethal damage) to the surface of the copper coupons. The cells were removed as described above, and spread on solidified minimal medium containing glycerol and 20 μL/mL D-cycloserine to select for *cycA* mutants. After 24 h of incubation, we counted the number of bacterial colonies that were considered to originate from the mutation of the *cycA* gene. The percentage of *cycA* mutants was calculated based on the ratio of the number of CFU of *cycA* mutants to the number of CFU of total bacterial cells. In the control treatment, bacterial cells were exposed for the same period of time to stainless steel or stainless steel in the presence of 0.25% (wt/vol) formaldehyde.

### 3.5. Single Cell Gel Electrophoresis (SCGE)

Comet assay or SCGE was performed as described by Sing *et al.* [25] with some changes. Suspensions of *B. glumae* strain AU6208 at the concentration of 5 × 10^7^ CFU/mL were streaked on dry copper surfaces using sterile cotton swabs. Cells were removed with 10 mL PBSG containing 20 µM EDTA, treated with lysozyme (20 mg/mL), and incubated at 37 °C for 20 min. Cells were then mixed with 0.8% agarose and applied to glass slides pre-coated with 1.5% agarose. After gelling and solidification of agarose cell suspensions, slides were immersed in lysis buffer (2.5 M NaCl, 0.1 M EDTA, 10 mM Tris-HCl, pH 10, 10% Triton X-100, and 1% dimethyl sulfoxide (DMSO) to prevent oxidation during lysis) and were carefully agitated at 25 rpm at 4 °C for 5 min. After washing with deionized water, slides were treated with denaturation buffer (300 mM NaOH and 1 mM EDTA, pH > 13), incubated briefly with excess Tris-borate-EDTA (TBE) buffer, and subjected to electrophoresis at 25 mV, 15 mA for 3 min. Slides were removed, washed with ice-cold deionized water, immersed into absolute ethanol, and air dried overnight. Finally, slides were stained with ethidium bromide (Sangon Biotech, China) in TBE and incubated for 1 min in the dark. Fluorescence was then observed (excitation wavelength [λ_Ex_] of ~490 nm, emission wavelength [λ_Em_] of ~520 nm) with an inverted confocal fluorescence microscope (Olympus DP50 BX 51).

### 3.6. ICP-MS Analysis

The influx of copper ions into cells of *B. glumae* strains AU6208 and LMG2196 was analyzed using inductively coupled plasma mass spectroscopy (ICP-MS) (Agilent ICP-MS model 7500a). *B. glumae* cells were exposed to copper and PVC surfaces for 3 h as described in the viability assays. Surface challenged cells on moist copper surfaces and PVC were removed and washed with ice-cold PBSG buffer containing 20 µM EDTA, and viable bacterial counts were determined by plating as described above. In parallel, samples were mineralized with concentrated 70% (v/v) nitric acid for 2 h at 70 °C and diluted to a final concentration of 5% (v/v) nitric acid. Germanium as Ge(NO_3_)_3_ was added at a final concentration of 50 ppm as an internal standard. Samples were loaded using an auto sampler and then analyzed. Each sample contained three replicates.

### 3.7. Membrane Damage

Damaged and intact membranes were observed in bacterial cells exposed to copper and control surfaces based on live/dead (*Bac*Light bacterial viability kit; Invitrogen) staining technique. This kit contains two nucleic acid stains, propidium iodide stain (a red-fluorescent), and SYTO 9 stain (a green-fluorescent). Cells were applied to and removed from surfaces as described above. The staining procedure was performed according to the manufactures instructions (*Bac*Light bacterial viability kit; Invitrogen). Briefly, suspensions of strained cells were incubated in the dark for 15 min and then transferred onto glass slides. Fluorescence was then observed (excitation wavelength [λ_Ex_] of ~490 nm, emission wavelength [λ_Em_] of ~520 nm) with an inverted confocal fluorescence microscope (Olympus DP50 BX 51).

### 3.8. Statistical Analyses

The data were subjected to analysis of variance, and the mean values were compared using the least significant differences test.

## 4. Conclusions

Taken together, our study demonstrated that copper has strong antibacterial activity against both strains of *B. glumae*, which is an emerging infectious agent found in a variety of environmental niches, plant rhizosphere, soil, hospital patients, and industrial contaminants. To the best of our knowledge, this is the first study revealing the antibacterial effects of copper against the *B. glumae* species. Furthermore, the results revealed that the killing is mainly due to a loss of cell membrane integrity and copper accumulation in bacterial cells, suggesting membrane proteins and lipids may be a major target of copper surface toxicity. Overall, these findings demonstrated the strong antibacterial activity of copper contact killing against multi-drug resistant *B. glumae* and its general mode of action in particular the role of membrane damage in bacterial killings.
